# Trade or a Learned Profession?

**Published:** 1913-12-20

**Authors:** G. Percy Joy


### The National Medical Union.

### Trade or a Learned Profession?

*To the Editor of* The Hospital.

Sir,—If conviction can be brought home to men of character in the profession that the National Medical Union intends to fight for principles, the success of the Union is assured.

In your issue of December 13 you have stated the true line of cleavage in the profession—the line between those who conform to professional standards and those who practise medicine as a trade.

Let the National Medical Union stand for an honourable profession, and let its appeal be (i) to the nation, in the interests of the common weal; and (ii) to the profession, in the interests of its honour and its power for service. Such a policy must involve (*a*) adequate remuneration and (*b*) effective control; the former entails the fixing of minimum fees, and the latter must be exercised in regard to such practices as undercutting, advertising, and varying the quality of professional services with the fee paid (for sweated labour, bluff, and the rendering of less than the best of which one is capable, are all dishonourable).

I submit to you, Sir, that a definite policy on these lines must win success, or there will emerge two classes of registered practitioners, the professional and the unprofessional.

If one can conceive that the public will be content to be at the mercy of the latter, these gentry may be relied on to see that their patients get no more than the price will fetch; assuredly their trade will be made to pay them at whatever price. Non-panel men have earned the right to lead on these lines; let them be ready and even eager to extend the right hand of fellowship to those on the panel who are willing to follow the lead.

As to the National Insurance Act as at present administered—Sir, we were made upright, let us leave this damnable invention to those whose oblique minds rejoice in it; let us stand upon our feet.—I am, Sir, yours truly,

G. Percy Joy.

